# Transgenic plants as a sustainable, terrestrial source of fish oils

**DOI:** 10.1002/ejlt.201400452

**Published:** 2015-06-23

**Authors:** Johnathan A. Napier, Sarah Usher, Richard P. Haslam, Noemi Ruiz‐Lopez, Olga Sayanova

**Affiliations:** ^1^ Department of Biological Chemistry and Crop ProtectionRothamsted ResearchHarpendenUK; ^2^ Instituto de la Grasa (CSIC)SevilleSpain

**Keywords:** Aquaculture, GM plants, Omega‐3, Plant biotechnology

## Abstract

An alternative, sustainable source of omega‐3 long chain polyunsaturated fatty acids is widely recognized as desirable, helping to reduce pressure on current sources (wild capture fisheries) and providing a de novo source of these health beneficial fatty acids. This review will consider the efforts and progress to develop transgenic plants as terrestrial sources of omega‐3 fish oils, focusing on recent developments and the possible explanations for advances in the field. We also consider the utility of such a source for use in aquaculture, since this industry is the major consumer of oceanic supplies of omega‐3 fish oils. Given the importance of the aquaculture industry in meeting global requirements for healthy foodstuffs, an alternative source of omega‐3 fish oils represents a potentially significant breakthrough for this production system.

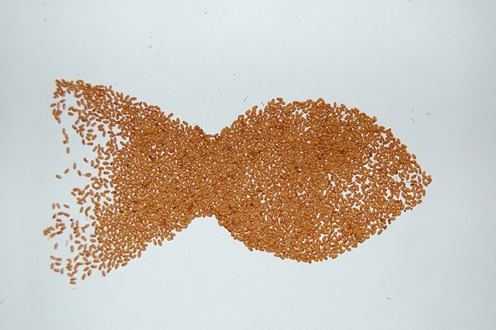

Transgenic Camelina seeds engineered to accumulate the omega‐3 fatty acids EPA and DHA, represent a sustainable alternative to fish oils.

**Abbreviations**
DHA: docosahexaenoic acid
EPA: eicosapentaenoic acid
GM: genetically modified
LC‐PUFAs: long chain polyunsaturated fatty acids

## 1 Introduction

The aquaculture (fish farming) industry is heavily dependent on fish oil as a commodity ingredient for the formulation of fish feeds—this is based on the nutritional requirements of marine and salmonid species for omega‐3 long chain polyunsaturated fatty acids (LC‐PUFAs) in their diets, and also to ensure that the final product destined for the consumer contains these health beneficial fish oils 1. Counter‐intuitively, most farmed fish species are unable to synthesise omega‐3 fish oils such as eicosapentaenoic acid (EPA) and docosahexaenoic acid (DHA), and are entirely dependent on dietary sources 2. Thus, the predominant farmed species such as salmon and trout represent major consumers of oceanically‐sourced fish oils (harvested from the so‐called reduction fisheries containing species such as anchovies, menhaden, and capelin), and collectively the aquafeed industry consumes in excess of 7 50 000 metric tons of fish oils per annum 3. When this demand is overlaid with the continuing expansion of fish‐farming (on average 6% per annum for the last two decades) 4 and the ever‐growing human population, there are a number of significant issues. Firstly, fish‐farming is the most effective system for producing animal protein for human consumption—the input/output ratios for aquaculture are superior compared to all terrestrial animal production systems 5. Secondly, farmed fish is now near‐ubiquitous in many countries—for example, 95% of all salmon sold in the UK is farmed 6. Thirdly, the finished products have well‐defined levels of health‐beneficial omega‐3s 7, though feed inclusion rates are showing a downward trend, most likely as a consequence of price‐sensitivity for raw materials such as fish oil 6, 8. Thus, all of these factors need to be considered in the continued attempts to ensure that fish farming remains an economically‐viable and environmentally‐sustainable means by which to ensure human health and nutrition. However, the continued expansion of the aquaculture industry, and its ability to deliver a nutritionally—important product, are constrained by the sustainable availability of omega‐3 fish oils with which to formulate feeds. Reduced wild fish stocks and associated quotas to protect these limit the annual global harvests of fish oils to ∼1 million metric tons, of which <20% is used for direct human nutrition 7. Thus, the limited availability of fish oils represents the critical bottleneck in this production system, and one that all major aquafeed companies in this field are trying to overcome. Attempts to replace fish oils in aquafeed diets with conventional vegetable oils (which are devoid ofthe critical fatty acids such as EPA and DHA) result in finished products that lack the health‐beneficial omega‐3 fish oils and run the risk of losing consumer‐confidence in oily fish as a healthy foodstuff 8. It is for all of these reasons that we set out to develop a new, sustainable, source of fish oils via their de novo synthesis in transgenic (genetically modified; GM) plants. This review will consider the approach we have adopted, contrast other alternative systems, and also examine the steps that still need to be taken to ensure that this research is translated into a tangible outcome.

## 2 Context and progress to date

The accumulation of LC‐PUFA EPA and DHA in the marine environment is predominantly via the biosynthetic activities of unicellular organisms such as microalgae, diatoms, and some bacteria; organisms that form the base of the marine foodweb. In general, most trophic levels above these microbes have limited to zero capacity to make EPA and DHA, most likely as a consequence of their environments being so omega‐3 rich and hence providing a continuous dietary source.

**Figure 1:**
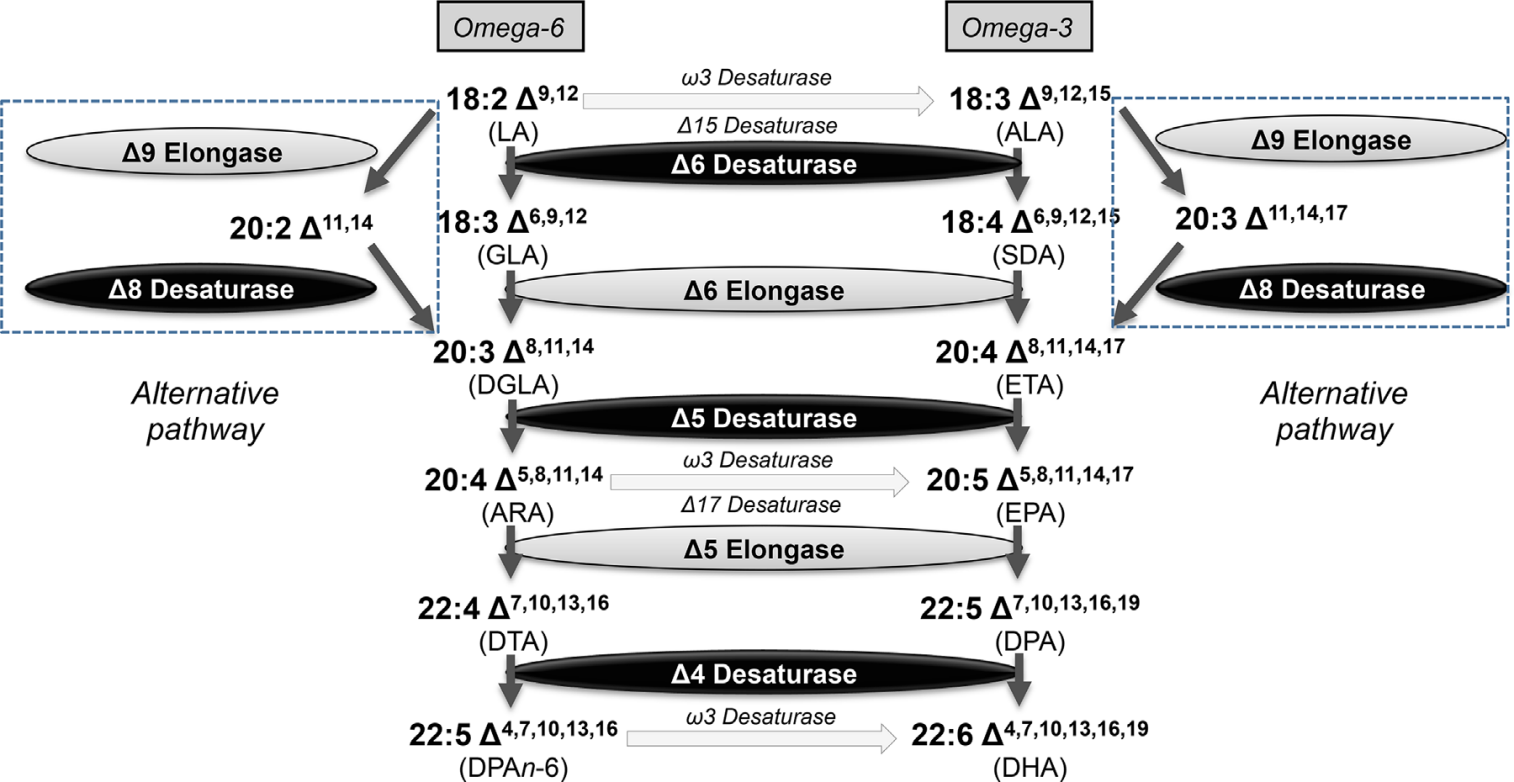
Schematic representation of the aerobic pathways for the biosynthesis of omega‐3 long chain polyunsaturated fatty acids. The relevant desaturase and elongase activities, and their associated substrates, are indictated, as is the variant form (the alternative pathway) observed in some limited examples.

Several different biosynthetic pathways for EPA and DHA have been identified in marine microbes, and the biochemical and molecular nature of these have been well described previously 9, 10, 11, but for clarity we will summarize these briefly. The predominant route by which microalgae and diatoms synthesise omega‐3 LC‐PUFAs is via a series of sequential desaturation and elongation reactions—this is an aerobic process, and can be further sub‐divided into two forms—the predominant “Δ6‐pathway,” in which the first committed step is the introduction of a Δ6‐desaturation into a C18 substrate, followed by C2 elongation and further Δ5‐desaturation. The much less common route, the so‐called alternative or “Δ8‐pathway,” initiates with the C2 elongation of the C18 substrate, followed by two successive desaturation reactions (Δ8, Δ5) 12. If DHA is also synthesized, this occurs by the C2 elongation of EPA and further (Δ4) desaturation. These two pathways are represented schematically in Fig. 1. Multiple genes for all of these activities have been identified in the last decade from a range of different microbial sources.

An entirely different route favored by some bacteria and a few examples of unicellular marine eukaryotes is the biosynthesis of EPA or DHA via an anaerobic pathway which uses a processive polyketide synthase‐like enzymatic system to convert malonyl‐CoA to omega‐3 LC‐PUFAs without any fatty acid intermediates. The genes for this pathway have also been identified and functionally characterized 13.

Collectively, all these different genes and the enzymatic activities they encode, represent a toolkit by which the metabolic engineer/synthetic biologist can attempt to reconstitute the capacity to synthesise EPA and DHA in a heterologous host. However, this is not as straight‐forward as it might appear, for a number of reasons. Firstly, in the case of the aerobic pathways, the substrate for the constituent enzymes is pre‐existing C18 di‐ or tri‐unsaturated fatty acids. In the case of the anaerobic PKS‐like pathway, the substrate is malonyl‐CoA, which is a low‐abundance primary metabolite. Secondly, the successful reconstitution of the complete pathway requires the coordinated expression and activity of multiple genes, which is non‐trivial in many transgenic systems. Thirdly, by definition, the host into which one is introducing the capacity to synthesise EPA and DHA has limited (or no) endogenous capability to make these fatty acids, meaning that native enzymes and pathways will be unfamiliar with these fatty acids—this could result in either selective exclusion of EPA/DHA from desired pathways (such as neutral lipid synthesis) or alternatively, the “trapping” of biosynthetic intermediates in undesired metabolic dead‐ends through the inclusive promiscuous activity of house‐keeping lipid enzymes. Finally, the underlying biochemical structural and biophysical properties of these enzymes are only just starting to emerge, mainly due to the membrane‐associated nature which precludes crystal structure‐based studies 14. Thus, although the genes with which to attempt the heterologous expression of omega‐3 LC‐PUFA biosynthetic pathways have been in hand for over 10 years, the first successes are only now beginning to emerge 15, 16.

In the context of the focus of this review, we will mainly consider the potential of genetically engineered oilseed crop plants to produce EPA and DHA, since it is likely that only such agricultural systems will have the capacity (in terms of scalability) to produce, in an economically viable manner, sufficient omega‐3 LC‐PUFAs for “high volume/low value” markets such as the aquafeed industry. However, we will also briefly consider attempts to produce these fatty acids in transgenic yeasts and diatoms, and how these contrast with the situation observed in plants.

### 2.1 Engineering the accumulation of EPA and DHA in transgenic plants—first attempts

As mentioned above, examples of all the biosynthetic genes required for the accumulation of EPA and DHA biosynthesis were identified by 2004 9, 17, facilitating their combined introduction into transgenic plants. Prior to that, individual genes from this pathway had been evaluated in plants, notably the Δ6‐ and Δ5‐desaturases—these data gave confidence that such heterologous activities were active in plant cells, pointing the way for subsequent endeavors 18. The first successful (proof‐of‐concept) demonstration of the feasibility of making EPA in a transgenic plant was published by Qi et al 19, expressing algal components of the alternative pathway in the leaves of Arabidopsis. Interestingly, this not only generated moderate amounts of EPA, but also the C20 omega‐6 LC‐PUFA arachidonic acid (ARA). Although this study was the first to generate EPA in a transgenic plant, the approach taken was via sequential transformation of individual genes, which was less than optimal (since it means that the three transgenes are genetically unlinked and prone to segregation in subsequent generations). Moreover, although vegetative accumulation of EPA was achieved, this was predominantly in phospholipids, whereas the target lipid species would ideally be neutral storage lipids such as triacylglycerols found in seeds.

The first demonstration of the seed‐specific accumulation of EPA was also achieved in the same year by Abbadi et al. 20, who expressed genes of the conventional Δ6‐pathway in transgenic linseed, under the control of seed‐specific promoters. Analysis of the resulting transgenic plants and their seeds confirmed the presence of low levels of EPA, but also very high levels of C18 Δ6‐desaturation products. This lead the authors to hypothesize that this unwanted build‐up of a biosynthetic intermediate was as a consequence of poor acyl‐exchange between different metabolic pools, and this concept has been further defined as “substrate dichotomy” 18. In this situation, a bottleneck is generated as a result of the differing substrate requirements for the sequential reactions present in the aerobic biosynthesis of LC‐PUFAs—specifically, most lower eukaryotic desaturases require their fatty acid substrate to be linked to a glycerolipid backbone, whereas fatty acid elongases require the substrate in the form of acyl‐CoAs.

**Figure 2:**
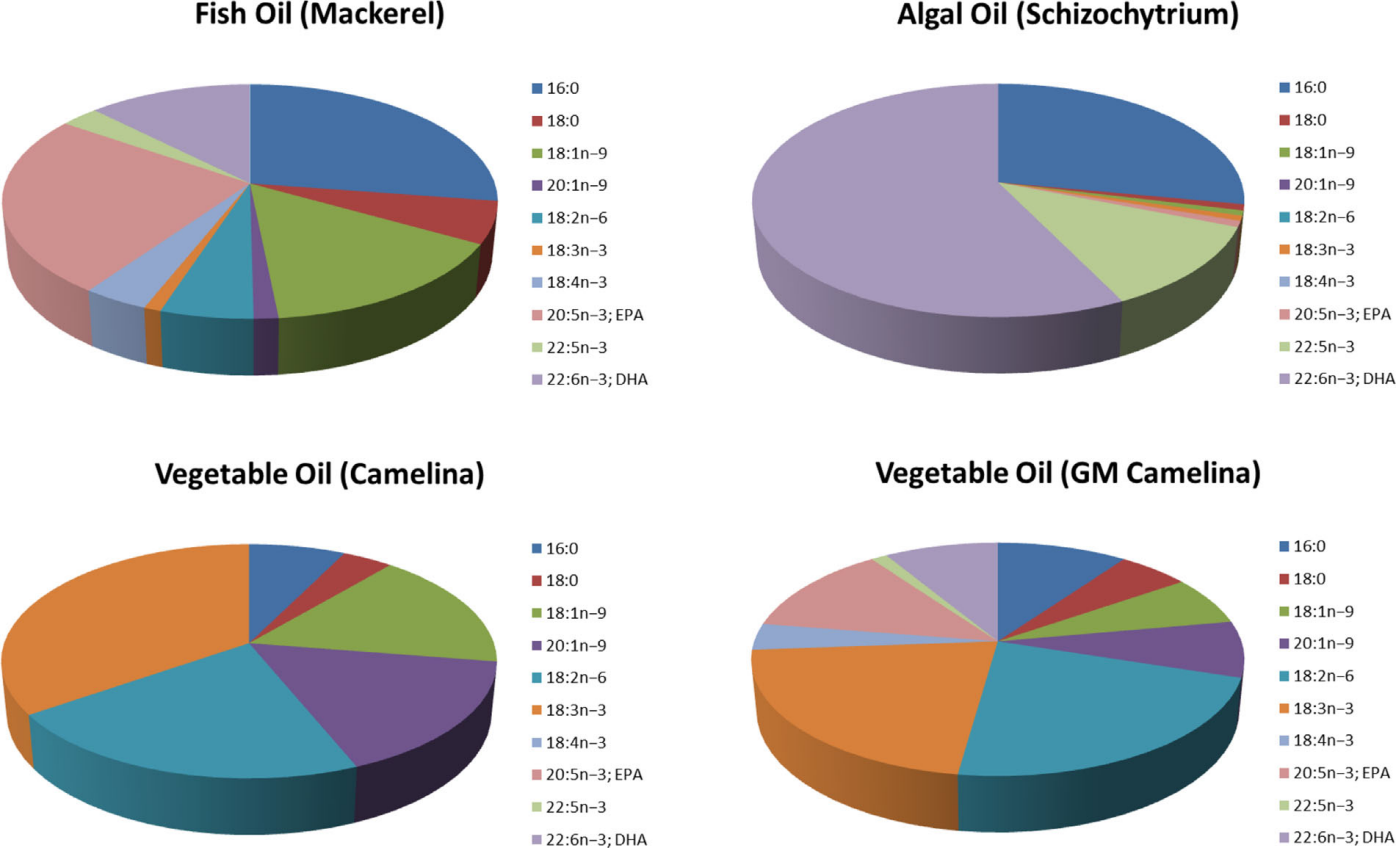
Comparison of fatty acid composition in different sources of omega‐3 LC‐PUFAs. The major fatty acids present in either fish oil, vegetable oil, GM vegetable oil, or algal oil are presented. Note that non‐GM vegetable oil lacks any EPA and DHA.

These initial first steps formed the basis for further iterations to drive up the accumulation of target fatty acids (EPA, DHA) and reduce the levels of undesired biosynthetic intermediates (such as the Δ6‐desaturation product γ‐linolenic acid; GLA). These studies have confirmed first the ability to make significant levels of EPA, albeit with attendant levels of GLA, 21, 22 and then also DHA 21, 23. The critical breakthroughs in optimizing the accumulation of target fatty acids were achieved by: (i) the use of omega‐3 specific desaturases, which prevented the accumulation of unwanted omega‐6 fatty acids; (ii) the use of acyl‐CoA‐dependent desaturases for the first committed (Δ6) step on the pathway 24, breaking the substrate‐dichotomy bottleneck and reducing the accumulation of GLA 22, 24, 25, 26, 27. As a consequence, it is now technically possible to accumulate fish oil‐like levels of omega‐3 LC‐PUFAs in the seed oils of transgenic plants similar to that found in fish oils, in which EPA and DHA accumulate to ∼20% of total fatty acids. A comparison of the fatty acid composition of fish oil, vegetable oil (GM and native), and also algal oil is shown in Fig. 2.

### 2.2 Approaches to successful accumulation of target fatty acids

Two discrete paths were adopted to ultimately produce EPA and DHA in the seeds of transgenic plants, though both started from the same position of having a “toolkit” of suitable biosynthetic genes isolated from microalgae and other omega‐3 accumulators, validated by heterologous expression in yeast. The first approach, exemplified by Petrie and colleagues at CSIRO (Canberra, Australia), took advantage of *Agrobacterium*‐mediated transient expression systems which allow the rapid evaluation of multiple gene combinations (via co‐infection) in the leaves of *Nicotiana* *benthamiana* 28. This allowed the identification of an optimal set of non‐native genes which directed the efficient synthesis of EPA and DHA in plants, albeit non‐seed tissues. However, by the further co‐expression in leaves of a “master regulator” transcription factor *LEC2*, the authors were able to “reprogramme” lipid metabolism in this tissue so that it now more closely resembled that of seeds, with the specific capacity to synthesize seed oil in the form of triacylglycerol. Thus, the host leaf cells under evaluation came to more closely resemble the metabolic context observed in seeds. In addition, an added benefit of this transcription factor‐mediated reprogramming was that instead of having to use constitutive promoters to drive the expression of the non‐native genes, it was now possible to use seed‐specific promoters (since they were now active as a result of the *LEC2* expression), meaning that both regulatory elements and biosynthetic enzymes were validated in a plant host. The resulting study 29 allowed for the selection of the optimal cassettes for subsequent stable transformation into plants (discussed below).

The alternative approach, adopted by ourselves and colleagues at the University of Hamburg, Germany was to directly assess and iterate gene combinations and their associated regulatory elements in the seeds of stably transformed transgenic plants 20, 21. The first example of this 21, demonstrated the potential of this approach, although the resulting seed oil profile still was suboptimal. Using transgenic Arabidopsis, we subsequently systematically evaluated 22 genes in 9 different combinations, and in 2 different genetic backgrounds 30, 31. For each construct, multiple independent transgenic lines were subject to detailed lipidomic analysis to define the incorporation of non‐native fatty acids into different lipid classes, which was then used to inform subsequent iterations. This systematic approach also identified an optimal suite of (different) genes for introduction into crop species 30, 31.

### 2.3 The transition from model to crop

As indicated above, different approaches resulted in the identification of a combination of algal and other lower eukaryotic activities which were capable of directing the synthesis and accumulation of EPA and DHA in model systems such as Arabidopsis or tobacco. However, this was still one step removed from the demonstration of the accumulation of these fatty acids in a suitable oilseed crop plant. Given the significant volumes of fish oils being annually removed from the oceans, it is only via the use of agricultural crops (and the scalability that brings) can one hope to address the challenges associated with generating a potential substitute.

To date, two oilseed crop species have been identified as potential hosts for the omega‐3 LC‐PUFA biosynthetic trait—canola, a cultivar of rapeseed (*Brassica napus* L.) and (*Camelina sativa*). In the case of canola, there are no peer‐reviewed data yet in the public domain to allow an accurate assessment. However, for Camelina, both the Rothamsted and CSIRO groups have demonstrated the suitability of this crop to act as an efficient host for the accumulation of EPA and DHA, although interestingly, the fatty acid profile obtained varies between the two studies. In the first publically available data 32, we built on our iterative studies in Arabidopsis to test two optimized constructs in transgenic Camelina—one directing the synthesis of just EPA, and one making both EPA and DHA. Based on the total fatty acid composition of seeds from stably transformed lines, the average level of EPA alone was 24%, and for EPA and DHA, 11 and 8%, respectively 32. Very low levels of undesired C18 biosynthetic intermediates such as GLA and stearidonic acid were observed. Interestingly, for both the EPA and EPA+DHA lines, the endogenous levels of α‐linolenic acid (ALA) were markedly decreased (presumptively since this fatty acid serves as direct precursor for the synthesis of EPA), whereas linoleic acid was effectively unchanged, most likely due to the transgene presence of a FAD2 Δ12‐desaturase. Surprisingly, levels of oleic acid were also strongly reduced for both constructs. Overall, the total level of target omega‐3 LC‐PUFAs was 19% (EPA+DHA), though this was found to be greater than 25% in some single seeds.

In the CSIRO study 33, a construct (GA7) previously evaluated in Arabidopsis (Petrie et al., 2012) was introduced into Camelina, and the performance compared with two additional constructs (mod‐F, mod‐G) in which minor changes were made. Significant levels of DHA (up to 12.4% for mod‐F) were obtained for all three constructs, though the levels of EPA were markedly low (maximally 3.3% for mod‐F). In contrast to the results observed by Ruiz‐Lopez et al., 32, levels of ALA were only moderately reduced, whereas LA was down from 18.1 to 8.4% (mod‐F), whereas accumulation of the C18 Δ6‐desaturation product stearidonic acid was noticeably higher. The observed differences between the various constructs almost certainly resulted from the contribution of a number of different factors, including but not limited to: (i) precise expression of seed‐specific promoters; (ii) superior examples of the same enzyme activity; (iii) construct design and orientation of elements; and (iv) site of integration in the Camelina genome. Perhaps the most striking and relevant difference between the two studies is the variation in the level of EPA—although the levels of DHA are broadly comparable (>10%), Petrie et al. 33 reported only 0.8–3.3% EPA for the three constructs, in contrast to the >11% observed by Ruiz‐Lopez et al. 32. The most likely explanation for this is the use of a highly active Δ5‐elongase in the former study, which delivers increased levels of DHA at the expense of EPA.

One additional interesting observation from these studies relates to the apparent utility (or not) of using a model system to predict the subsequent outcome in crop systems. Based on both sets of results obtained in Camelina, it appears as though previous studies in Arabidopsis underestimated the levels of target fatty acids obtainable in the crop, though obviously these earlier studies formed an important part of the proof‐of‐concept. For example, in the earlier Arabidopsis study by Ruiz‐Lopez et al. 30, 31, maximal levels of EPA observed were ∼8%, whereas in Camelina this was 24% 32. Similarly, Petrie et al. 23 reported an average of 5.5% DHA in T2 Arabidopsis seeds, compared with 9.6% DHA in T2 Camelina seeds 33. Bearing in mind that in both studies, the same identical constructs (EPA‐A5.1; GA7, respectively) were introduced into the two different hosts, these data would imply that, at least for the omega‐3 LC‐PUFA trait, Arabidopsis may underestimate the outcome in more complex (genomically and biochemically) crops. In that respect, it may be that in addition to the four influencing factors described above, an additional consideration should be the “metabolic context” into which the heterologous pathway is superimposed 34. Since the transgene‐derived activities need to biochemically integrate with endogenous lipid metabolism to reconstitute the efficient synthesis of EPA and DHA, it may be beneficial to have multiple copies of such native genes, each with perhaps minor differences in expression pattern, substrate‐specificity, or efficiency. Thus, working in a hexaploid crop such as Camelina provides more opportunities (as a result of increased variation via homeologues) for the smoother incorporation of the heterologous pathway, and hence, higher synthesis and accumulation of target fatty acids.

### 2.4 Utility and application of novel plant oils

The availability of these modified Camelina oils is a very recent development, so there is currently only limited data on their properties and applications of them. However, two studies are of note. Firstly, Mansour et al. 35 examined the detailed composition of Camelina oil derived from the GA7 construct, looking at both glycerolipid composition and also sterols. Interestingly, the maximum level of DHA present in TAG (the dominant neutral lipid) was 6.8%, and basal levels of EPA (0.4%). There was significantly less DHA (1.6%) in phospholipids, though the residual protein meal also contained DHA (5.4%) though the overall lipid content in this fraction was very low (0.3% of starting seed weight). Although this preliminary study did not indicate which generation or event for the GA7 construct was examined, it is noteworthy that the levels of DHA in TAG are somewhat reduced compared to the original study (6.8% vs. 9.6%; respectively) 33, 35. There was no significant difference between the GA7 line and wildtype Camelina with regards to levels or profiles of sterols, indicating that the presence of non‐native omega‐3 LC‐PUFAs did not alter this pathway.

More recently, the first study on the application of these novel oils has been published—Betancor et al. 36 used the EPA‐containing Camelina oil described by Ruiz‐Lopez et al. 32 to determine if this oil could substitute for fish oil in a salmon feeding trial. In this study, juvenile (post‐smolt) Atlantic salmon were fed diets containing fish oil, EPA‐containing Camelina oil, or wild type Camelina oil. All three diets were isocalorific, and also contained low levels of DHA (via the deliberate use of fish meal as a protein source) to ensure no deficiency for this fatty acid. The fish were fed the different diets for seven weeks, after which they had all doubled in weight and displayed no indicators for ill‐health. Fatty acid analysis of the flesh and the liver indicated the expected accumulation of EPA, and in the latter tissue, some conversion to DHA 36. Transcriptomic analyses of these tissues also indicated the up‐regulation of genes involved in omega‐LC‐PUFA metabolism, though both Camelina treatments (WT, GM) induced transcriptome signatures more similar to each other than the fish oil treatment—this most likely reflects the fact that the GM oil still contains fatty acids normally associated with vegetable oils, but not observed in fish oils (such as linoleic acid), and also the common presence of (phyto) sterols, which are known to induce the up‐regulation of the cholesterol pathway in fish 37. In that respect, the EPA‐containing GM Camelina oil can be considered a “hybrid” oil, representing an in vivo blend of plant and marine fatty acids (see also Fig. 2 for graphical representation). Given that most aquafeed diet formulations are now a mixture of fish oil and vegetable oil, this GM Camelina oil is well‐suited for direct use in aquaculture. Moreover, this study, for the first time, demonstrates the utility of using novel oils derived from a GM plant as a safe and effective replacement for fish oils.

### 2.5 Alternative production systems for the synthesis of omega‐3 LC‐PUFAs

Currently, two additional approaches also contribute to the de novo production of omega‐3 LC‐PUFAs, via either the fermentation of a GM yeast strain, or via the cultivation of native strains of microalgae, and as such, may have the capacity to augment piscine sources of these fatty acids. In the latter case of algae cultivation such production systems use a diverse range of different algal strains, and play an important role in providing suitable nutrients to the earlier stages of the life cycle of various species used in fish‐farming, though the actual levels of omega‐3 LC‐PUFAs produced by this process are very modest (<1% of annual fish oil production rates). Currently, there are no examples of genetically modified algal strains being used in this way, though recently it has been reported that a GM strain of *Phaeodactylum tricornutum* had been produced which had increased levels of DHA 38.

In the case of microbial fermentation, work from DuPont demonstrated the feasibility of using the oleaginous yeast *Yarrowia lipolytica* as a heterologous host for the production of EPA. This tour‐de‐force of metabolic engineering introduced components of the alternative pathway into the yeast to enable it synthesize e EPA, with very high levels (up to 15% dry weight) of this fatty acid being obtained. Serendipitously, part of this high level of accumulation arose as a result of the insertional mutagenesis (via integration of the transgenes) of a gene (PXA10) involved in peroxisomal biogenesis, resulting in decreased levels of fatty acid catabolism by beta‐oxidation 39. The utility and applications of this source of EPA are discussed in a recent review 40.

## 3 Conclusions

As reviewed above, it is clear that the promise of a plant‐based source of omega‐3 LC‐PUFAs has now been deomonstrated, at least in the case of Camelina. However, there still remain a number of hurdles to clear before such a crop becomes a commercial reality and contributes to the pressing need for an alternative, sustainable source of omega‐3 LC‐PUFAs. Given the progressive and forward‐looking nature of the aquafeed industry, it is hoped that such novel ingredients can, subject to appropriate regulatory and safety approval, become incorporated into a wide range of fish diets, and in turn, relieve pressure on the oceanic stocks of the reduction fisheries. Since Camelina as an oilseed crop can easily yield 0.75 ton of oil/ha, then a GM oil containing similar levels of EPA and DHA to that found in fish oils could make a significant contribution to off‐setting oceanic sources. For example, 2 00 000 ha of GM Camelina could produce 1 50 000 MT of oil which could serve as a direct replacement for fish oils in aquafeed, representing 15% of the global oceanic harvest of these oils. And although 2 00 000 ha might seem like a large area, in agricultural terms, it is a quite modest scale—for comparison, the current annual Canadian sowing of related oilseeds such as canola is in excess of 7 million ha. Thus, the proposed 2 00 000 ha of Camelina represents only a small (<3%) area of land currently given over to vegetable oil production in one country, yet could significantly increase the de novo synthesis of omega‐3 LC‐PUFAs.

Converting this hypothetical concept into reality will require a number of different factors to be dealt with, including seeking and obtaining regulatory approval to grow a novel GM crop, better understanding of the agronomy and processing of Camelina, use and formulation of the novel oil by end‐users in the feed sector, and ultimately acceptance by the consumer. None of these steps are trivial or should be taken for granted, and will most likely require significant resources and organizational focus. However, given the increasing demand for omega‐3 LC‐PUFAs, and the demonstrated examples of “proof‐of‐concept” described in this article, it is hoped that a terrestrial, GM oilseed source of EPA and DHA might be available by the end of this decade.

4

*Rothamsted Research receives grant‐aided support from the Biotechnology and Biological Sciences Research Council of the U.K. Some of the work described in this review was supported by BBSRC grant BB/J00166X/1*.

*The authors confirm that they have no conflict of interest*.
